# Effects of Cordycepin on the Microglia-Overactivation-Induced Impairments of Growth and Development of Hippocampal Cultured Neurons

**DOI:** 10.1371/journal.pone.0125902

**Published:** 2015-05-01

**Authors:** Jie Peng, Ping Wang, Hongshan Ge, Xianqin Qu, Xingliang Jin

**Affiliations:** 1 Wuzhong Hospital, Suzhou, Jiangsu, China; 2 The Second Affiliated Hospital of Wenzhou Medical University, Wenzhou, Zhejiang, China; 3 Sydney Centre for Regenerative and Developmental Medicine, Kolling Institute for Medical Research, Sydney Medical School, University of Sydney, NSW, Australia; 4 School of Medical and Molecular Biosciences, University of Technology Sydney, Sydney, NSW, Australia; Indiana School of Medicine, UNITED STATES

## Abstract

Microglial cells are normally activated in response to brain injury or immunological stimuli to protect central nervous system (CNS). However, over-activation of microglia conversely amplifies the inflammatory effects and mediates cellular degeneration, leading to the death of neurons. Recently, cordycepin, an active component found in *Cordyceps militarisa* known as a rare Chinese caterpillar fungus, has been reported as an effective drug for treating inflammatory diseases and cancer *via* unclear mechanisms. In this study, we attempted to identify the anti-inflammatory role of cordycepin and its protective effects on the impairments of neural growth and development induced by microglial over-activation. The results indicate that cordycepin could attenuate the lipopolysaccharide (LPS)-induced microglial activation, evidenced by the dramatically reduced release of TNF-α and IL-1β, as well as the down-regulation of mRNA levels of iNOS and COX-2 after cordycepin treatment. Besides, cordycepin reversed the LPS-induced activation of NF-κB pathway, resulting in anti-inflammatory effects. Furthermore, by employing the conditioned medium (CM), we found cordycepin was able to recover the impairments of neural growth and development in the primary hippocampal neurons cultured in LPS-CM, including cell viability, growth cone extension, neurite sprouting and outgrowth as well as spinogenesis. This study expands our knowledge of the anti-inflammatory function of cordycepin and paves the way for the biomedical applications of cordycepin in the therapies of neural injuries.

## Introduction

Cordycepin, 3’-deoxyadenosine, an active component found in *Cordyceps militarisa* known as a rare Chinese caterpillar fungus, has been shown to be beneficial to circulatory, immune, respiratory and glandular systems in human body [1]. Given the biological effects including anti-fungal, anti-tumorigenic on some cell lines and anti-diabetic effects, cordycepin has been proposed to be used in the therapies against several diseases including inflammatory diseases and cancer [2–4]. Also, accumulating evidences suggested that cordycepin is able to affect central nervous system (CNS) and exert neuro-protective effects after cerebral ischemia or reperfusion injury [5, 6]. Recently, cordycepin was found to be capable of reducing excessive nitric oxide (NO), prostaglandin E_2_ (PGE_2_) and pro-inflammatory cytokine production, as well as inhibiting the eukaryotic transcription factor nuclear factor-kappa B (NF-κB) translocation by suppressing IkappaB-α (IκB-α) degradation, protein kinase B (Akt), ERK-1/2, JNK, and p38 kinase and mitogen-activated protein kinases (MAPK) signaling pathways to reduce lipopolysaccharide (LPS)-stimulated inflammation in murine BV2 microglia without causing cytotoxicity [7]. This finding provides a novel treatment for neurodegenerative diseases by repressing the productions of inflammatory mediators in microglia. However, the mechanisms of the anti-inflammatory effects of cordycepin on microglia still remain ambiguous.

Microglia is a type of immune cells in the CNS. These macrophage-like cells are normally activated in response to brain injury or immunological stimuli [8, 9]. Microglia has been demonstrated to participate in brain remodeling and maturation as it eliminates cells deemed to be cleared [10, 11]. Additionally, they also serve as immune surveillance and host defense protecting against invading microorganisms and neoplastic cells in the matured brain [12]. However, whether the activation of microglia plays a role in the neuronal death remains elusive since amplifying the inflammatory effects and mediating cellular degeneration in contrast to protecting CNS by activated microglia was observed. The activation of microglial cells with the dramatic up-regulations of their surface receptors promotes the secretions of a variety of soluble factors, including glia-derived neurotrophic factor that potentially assists the survival of neurons [13]. Simultaneously, it also facilitates the production of some proinflammatory and neurotoxic factors such as cytokines tumor necrosis factor-α (TNF-α) and interleukin-1β (IL-1β), free radicals, fatty acid metabolite and quinolinic acid [13].In turn, the excessive activated microglial cells induced by subtle changes in their micro-environment or pathological insults, can be deleterious to neurons and propagate neuronal injury [14, 15]. Uncontrolled activated microglia was proposed to have reduced capacity to protect neurons instead promote neurotoxity by releasing inflammatory mediators and cause bystander injury to neurons [12, 16]. In this case, the regulation of neuroinflammation by microglia functions as a double-edged sword in maintaining brain health [17]. Accumulating evidence linked neuroinflammation to the pathogenesis of several diseases such as Alzheimer’s and Parkinson diseases as well as several psychiatric disorders such as schizophrenia (SCZ) and depression [18–21]. Although the mechanisms of these diseases are still unclear, the overactivation of microglia is increasingly accepted as an important role in pathogenesis of these diseases.

In this study, we aim to investigate the neural protective effects of cordycepin against the impairments induced by microglial over-activation. We first studied the anti-inflammatory effects of cordycepin and the underlying mechanisms. Then we explored the beneficial effects of cordycepin on the impairments of neural growth and development in the primary hippocampal cultured neurons. The results revealed that cordycepin can alleviate the inflammation by microglial over-activation and protect the neurons against the following injuries.

## Materials and Methods

### Cell Culture

Microglia (BV2 cells) were purchased from the Shanghai Baili Biology Company (Shanghai, China). For the BV2 culturing, the cells were cultured in Dulbecco’s Modified Eagle Medium (DMEM; Gibco, USA) supplemented with 10% fetal bovine serum (FBS; Gibco), 100 U/mL penicillin and 100 mg/ml streptomycin (Gibco) at 37°C in a 5% CO_2_ humidified air environment. The different concentrations of cordycepin (1, 10, 20, 40 μg/ml) (Sigma, St. Louis, MO, USA) were added to the culture medium for the cordycepin treatment.

For the culturing of hippocampal neurons, the hippocampus of both hemispheres hippocampus was dissected from the brain of prenatal mouse. Cells were gently dissociated by pipette tips after digested in TryplE (Life Technologies, USA) for 15 min at 37°C. Cell suspension was then centrifuged at 1000 rpm for 5 min, and the pellet was resuspended in culture medium (DMEM medium containing 10% FBS, 2% B-27 supplement, penicillin-streptomycin, Ham’s F-12 Nutrient Mixture). Cultures were maintained at 37°C in humidified atmosphere with 5% CO_2_ and medium was changed 24 h after plating and every 3 days thereafter. To inhibit glial cell proliferation, on the second day we treated the culture with 5 μM cytosine-β-D-arabinofuranoside (Ara-C, Sigma). Neurons cultured for 7 to 21 days were used in the experiments. This study was carried out in strict accordance with the recommendations in the Guide for the Care and Use of Laboratory Animals of the National Institutes of Health. The protocol was approved by the Committee on the Ethics of Animal Experiments of Wuzhong Hospital. The IACUC committee members at Wuzhong Hospital approved this study. All surgery was performed under 0.3% sodium pentobarbital (1 mL/100g) anesthesia. The animals were sacrificed by CO_2_ inhalation followed by decapitation and all efforts were made to minimize suffering.

### Cell viability

Cell viability was measured by 3-(4,5-dimethylthiazol-2-yl)-2,5-diphenyl tetrazolium bromide (MTT) assay. Briefly, the cells were cultured in 96 well plates and the medium was removed and the cells were incubated with 0.5 mg/ml MTT solution for 4 h at 37°C. The supernatant was then discarded and the formed formazan crystals in each well were dissolved in DMSO (0.1%). The absorbance was measured at 490 nm with a Microplate reader (VictorTM ×4, PerkinElmer, Singapore). Cell viabilities were normalized to the control.

### LDH assay

The integrity of cells was evaluated by Lactase Dehydrogenase (LDH) assay following the protocol of CytoTox-ONE Homogeneous Membrane Integrity Assay kit (Promega, USA). The assays were performed after 7 days of culture. The data were recorded by a Multilabel Reader (Victor ×4, PerkinElmer, Singapore) and transparency characteristics of the substrates permitted an online observation of the cells with an inverse light microscope (Eclipse Ti-E, Nikon, Japan).

### Enzyme-linked immunosorbent assay (ELISA)

The concentrations of TNF-α and IL-1β in culture supernatants were measured by commercial ELISA Kit (Boster Biological Technology, China) according to the manufacturer’s instructions. The absorbance at 450 nm was determined using a microplate reader. The TNF-α and IL-1β concentrations in culture supernatants calculated using TNF-α and IL-1β standards.

### RT-PCR

Total RNA from cells were extracted by RNeasy Mini Kit (Qiagen, USA). Quality and quantity of RNA were measured by NanoDrop 8000 spectrophotometer (Thermo Scientific). 1000 ng RNA was used for each reaction to produce cDNA using high capacity cDNA reverse transcription kit (Applied biosystems) following manufacturer's instructions. cDNA products were diluted by adding RNase and DNase free water and frozen at -20°C before gene expression assay. Gene expression was measured with quantitative real-time RT-PCR system. The primers are as follow:

iNOS: sense 5′-GGACGAGACGGATAGGCAGAGATT-3′

antisense 5′-AAGCCACTGACACTTCGCACAA-3′;

COX-2: sense 5′-TCTCCAACCTCTCCTACTAC-3′

antisense 5′-GCACGTAGTCTTCGATCACT-3′;

β-actin: sense 5′-GATGGTGGGAATGGGTCAGA-3′

antisense 5′-TCCATGTCGTCCCAGTTGGT-3′;

Data analysis was performed using the 2^−ΔΔCt^ method.

### Conditioned medium (CM)

BV2 cells were first cultured for 24 h in the BV2 culture medium, then the cell culture supernatants were replaced by DMEM-F12 FBS-free medium with or without LPS stimulation in the absence or presence of cordycepin to culture another 24 h. CM were harvested, centrifuged for 5 min at 1000×g, filtered through 0.22-μm-pore-diameter Millipore filters, and then dialyzed overnight using the Slide-A-Lyzer Dialysis Cassette (10K MWCO, Pierce Biotechnology Inc., USA) to exclude the possible influence of LPS. CMs were stored at -80°C until use.

### Separation of Nuclei and Cytosol

Nuclei and cytosol were separated using a nuclear extract kit according to the manufacturer’s instructions (Active Motif).

### Immunostaining

The cells were treated following the protocols of the Fast ImmunoFluorescence Staining Kit (BPIF30-1KT, Protein Biotechnologies, USA). The primary antibodies used were as follows: anti-β-tubulin (Sigma), anti-actin (Sigma), MAP-2 (Sigma). For the measurement of the effect of cordycepin on LPS-induced inflammatory responses, nuclei were stained with DAPI after washing with phosphate-buffered saline, and fluorescence was visualized using a fluorescence microscope (Carl Zeiss, Oberkochen, Germany).

### Western blot

The expressions of p65, IкBα, p-IкBα and GAP43 were determined by western blotting and β-actin was employed as control while HDAC1 was used as a control of p65 in nucleus. Cell extracts were lysed in RIPA buffer (Beyotime, China) containing Complete Protease Inhibitor Cocktail and 2 mM PMSF. About 20 μg of total protein was separated by 10% SDS-PAGE and transferred to 0.45 μm Nitrocellulose Membrane (Millipore, USA). Protein concentrations were determined by BCA Protein Assay Kit (Beyotime). The membranes were incubated with primary antibodies at 4°C overnight and then hybridized with appropriate HRP-conjugated secondary antibody (Amersham Pharmacia Biotech) at room temperature for 2 h. The results were visualized using ECL kit (abcam) and observed by GeneGnome mechine (Syngene).

### Transfection

The cultured hippocampal neurons were transfected with mCherry-actin at day 17 by the calcium phosphate method and the images were taken at day 21.

### Statistical analysis

All the experiments were performed in triplicate and data are shown as mean ± SEM based on three separate experiments. Statistical analysis was performed using one-way ANOVA analysis followed by Tukey’s tests and p < 0.05 was considered as statistical significant.

## Results and Discussion

### Effect of cordycepin on microglial cell viability

**Fig 1:**
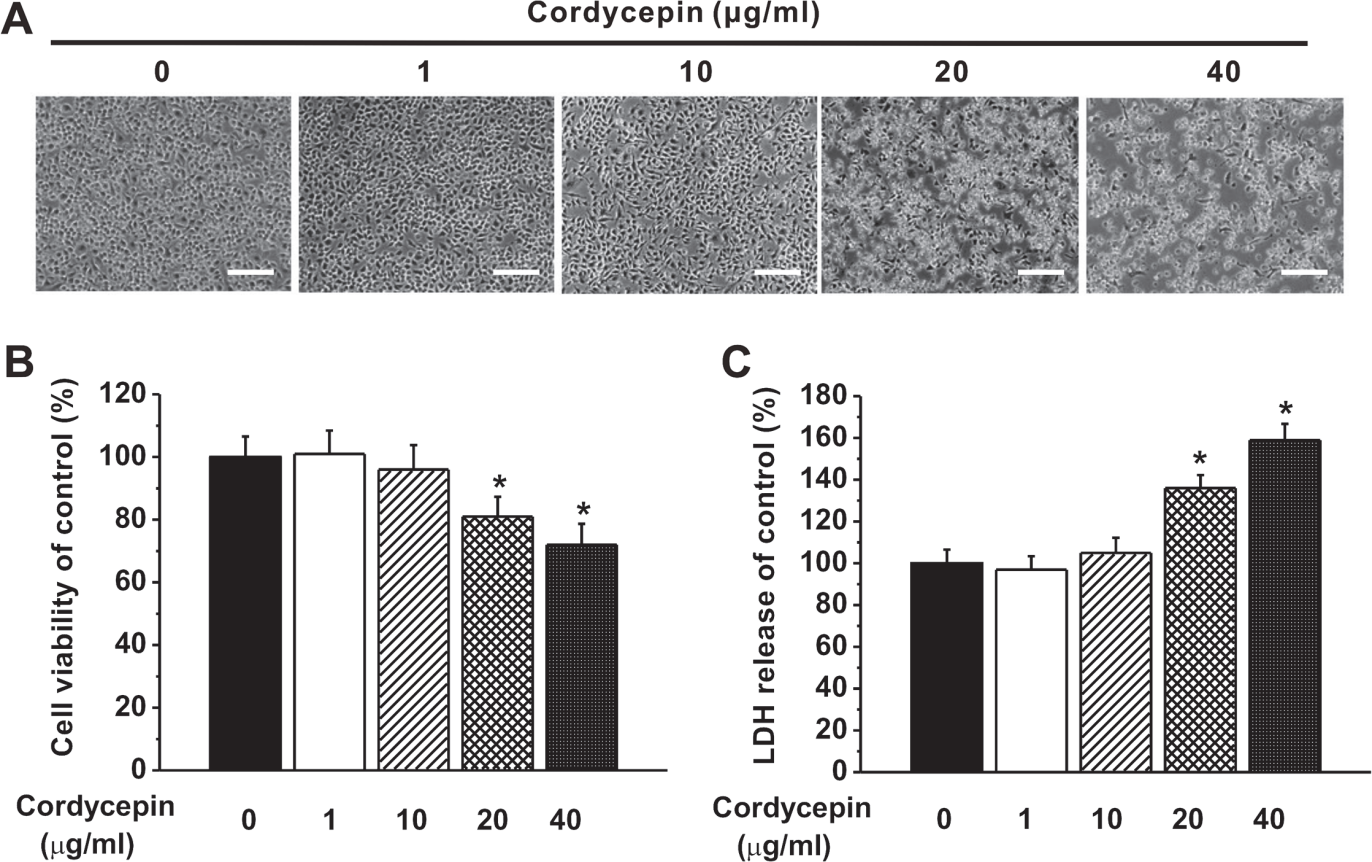
Effects of different doses of cordycepin exposure on BV2 microglia viability. (A) Representative photographs of microglia exposed to different doses of cordycepin (0, 1, 10, 20, 40 μg/ml). Scale bar = 200 μm. (B) Relative cell viability of the microglia to the control following cordycepin treatment, measured by MTT assay. (C) Relative LDH release to control in the experimental groups. The data were presented by the mean ± SEM. * p < 0.05.

Prior to investigating the possible anti-inflammatory effects of cordycepin, we initially determined the effect of cordycepin on the cell viability of the microglial cells (BV2 cells in this study). Four different concentrations of cordycepin (1, 10, 20 and 40 μg/ml) were employed, according to our preliminary experiments and previous reports that investigated the effects of different concentrations of cordycepin ranging from 5 to 40 μg/ml on cell viability [22, 23]. The cells under 1 and 10 μg/ml cordycepin treatment exhibited a similar cell density and morphology as those without cordycepin treatment (control) (Fig 1A). Significantly reduced cells were observed in the cultures with 20 or 40 μg/ml cordycepin treatments (Fig 1A). This was further validated by MTT assay and LDH release assay. No differences of cell viability were found between control, 1 and 10 μg/ml cordycepin treated groups, while cell viability were significantly decreased when treated with 20 or 40 μg/ml of cordycepin (Fig 1B). Also, LDH assays provided consistent result that cordycepin treatment with the concentrations less than 10 μg/ml had no obvious impairment on the integrality of the cell membrane (Fig 1C), as LDH release indicates membrane damage and is a hallmark of necrosis. High concentrations of cordycepin (20 or 40 μg/ml) could remarkably increase the LDH release compared to the control. Therefore, the concentration of 10 μg/ml was decided to be used as an optimized concentration for the following experiments.

### Cordycepin attenuated the LPS-induced microglial over-activation

**Fig 2:**
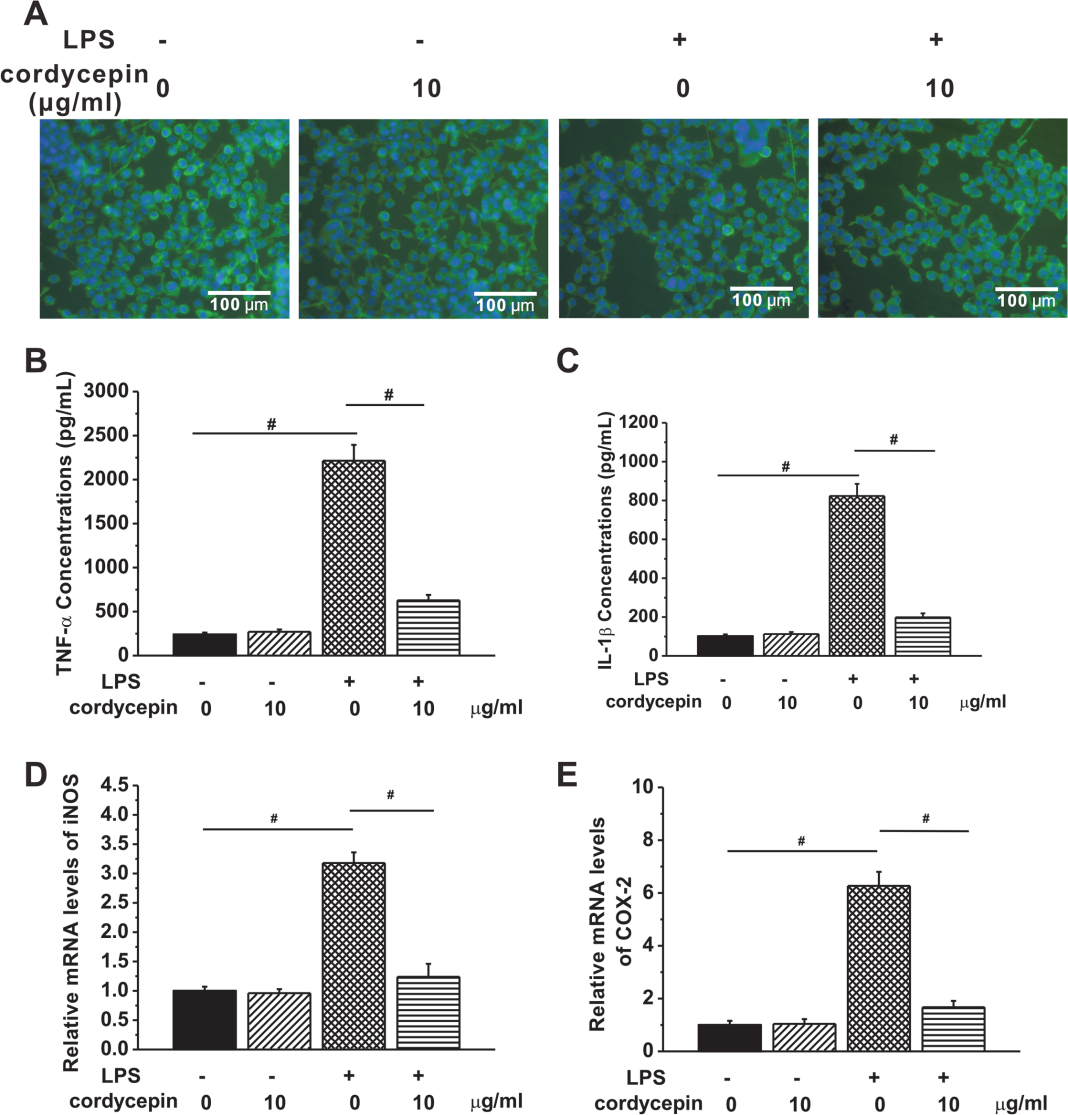
Cordycepin attenuated the LPS-induced inflammatory responses. (A) Representative images of microglia following 10 μM cordycepin treatment for 1 day with or without LPS treatment. Cells were stained green for tubulin, blue for nucleus. Scale bar = 100μm. The contents of cytokines, including TNF-α (B) and IL-1β (C) in the experimental groups were measured by the ELISA assay. Note the obviously lower concentrations of TNF-α and IL-1β in cordycepin treatment group compared to untreated group after LPS stimulation. (D-E) Relative mRNA levels of iNOS and COX-2 in the experimental groups assessed by real time PCR using the 2^-ΔΔCT^ method. Data are expressed as the fold change in gene expression normalized to an endogenous gene (β-actin) and relative to the cells in control group without LPS stimulation. Data are presented as mean ± SEM (n = 6 in triplicate). # p < 0. 01.

Microglia-mediated neurotoxicity has been most effectively demonstrated in models using LPS [24], the polysaccharide component of the cell walls of gram-negative bacteria. LPS can induce an obvious increase in the synthesis of inflammatory mediators, including various chemokines and cytokines [25, 26], which has been reported to affect the neural cell behaviors and functions [26, 27]. LPS was employed in our study based on two reasons. One is LPS-induced neuroinflammation could be a perfect positive control for us to evaluate the anti-inflammatory effects of cordycepin. The other is we can investigate whether and how the cordycepin treatment affects cell behaviors in the hippocampal neurons after microglia over-activation by LPS insult. To study the anti-inflammatory effects of cordycepin on microglia, microglia cells were cultured in the presence or absence of LPS (100 ng/ml, 24 h) with or without cordycepin treatment (10 μg/ml). The microglial cells in these groups demonstrated a variety of morphological characteristics, including amoeboid, spindle, ramified and spherical shapes, as shown in Fig 2A. We first examined the levels of two cytokines involved in the microglia induced inflammation in the experimental groups, tumor necrosis factor alpha (TNF-α) and interleukin-1 β (IL-1β). TNF-α is a key mediator of inflammation involved in the initiation, regulation and perpetuation of the inflammatory response [28]. Anti-TNF-α strategies have been applied as a clinical therapy for treating inflammatory diseases [29]. IL-1β is primarily a pro-inflammatory cytokine that can be rapidly up-regulated to defend against infection and injury [30]. No significant change was obtained in microglia without LPS stimulation. An increasing number of inflammatory diseases were found to be associated with the dysregulation of IL-1β activity [31, 32]. LPS significantly induced much higher levels of TNF-α and IL-1β release compared to control (Fig 2B and 2C). The treatments of 10 μg/ml of cordycepin on LPS-stimulated microglia led to significant decreases of TNF-α and IL-1β levels compared to the microglia with LPS stimulation (Fig 2B and 2C), suggesting that cordycepin could attenuate the microglial over-activation by LPS insult. Also, amyloid-beta (Aβ) was used to be another control to stimulate the microglia. The results show that Aβ can also induce microglial activation and cordycepin successfully reduced the Aβ-induced cytokine release (Fig. A in S1 File). Furthermore, we examined the expression of two important enzymes, inducible nitric oxide synthase (iNOS) and cyclooxygenase-2 (COX-2), which are involved in the microglia mediated inflammation. Inducible nitric oxide synthase (iNOS) plays a critical role in inflammation since it controls the production of nitric oxide (NO) [33]. Cyclooxygenase-2 (COX-2) can be massively detected at the site of inflammation as a key enzyme involved in the release of prostaglandins regulating inflammation [34, 35]. Given the improper elevation of iNOS and COX-2 occurring in several inflammatory disorders and their contributions to mediate inflammatory processes [36], we evaluated the mRNA expressions of iNOS and COX-2 in the cultures treated with cordycepin. No change was observed when microglia was normally activated, whereas dramatically increased mRNA expressions of iNOS and COX-2 were found in LPS-stimulated microglia. In the presence of 10 μg/ml of cordycepin, the mRNA levels of iNOS and COX-2 were both kept at control levels, further indicating that cordycepin suppressed the LPS-induced inflammation. These results are consistent with a previous study that showed 1h pre-treatment of cordycepin (1~7.5 μg/ml) was able to inhibit both pretranslational and translational levels of iNOS and COX-2 and mRNA expressions of TNF-α and IL-1β in LPS-stimulated BV2 microglia [7]. The transcription of iNOS and COX-2 can be activated by NF-κB, while TNF-α and IL-1β are involved in the activation of NF-κB pathway [36–39], implying a possible connection between the anti-inflammatory role of cordycepin and NF-κB signaling pathway.

### Cordycepin inhibited NF-κB signaling pathway

**Fig 3:**
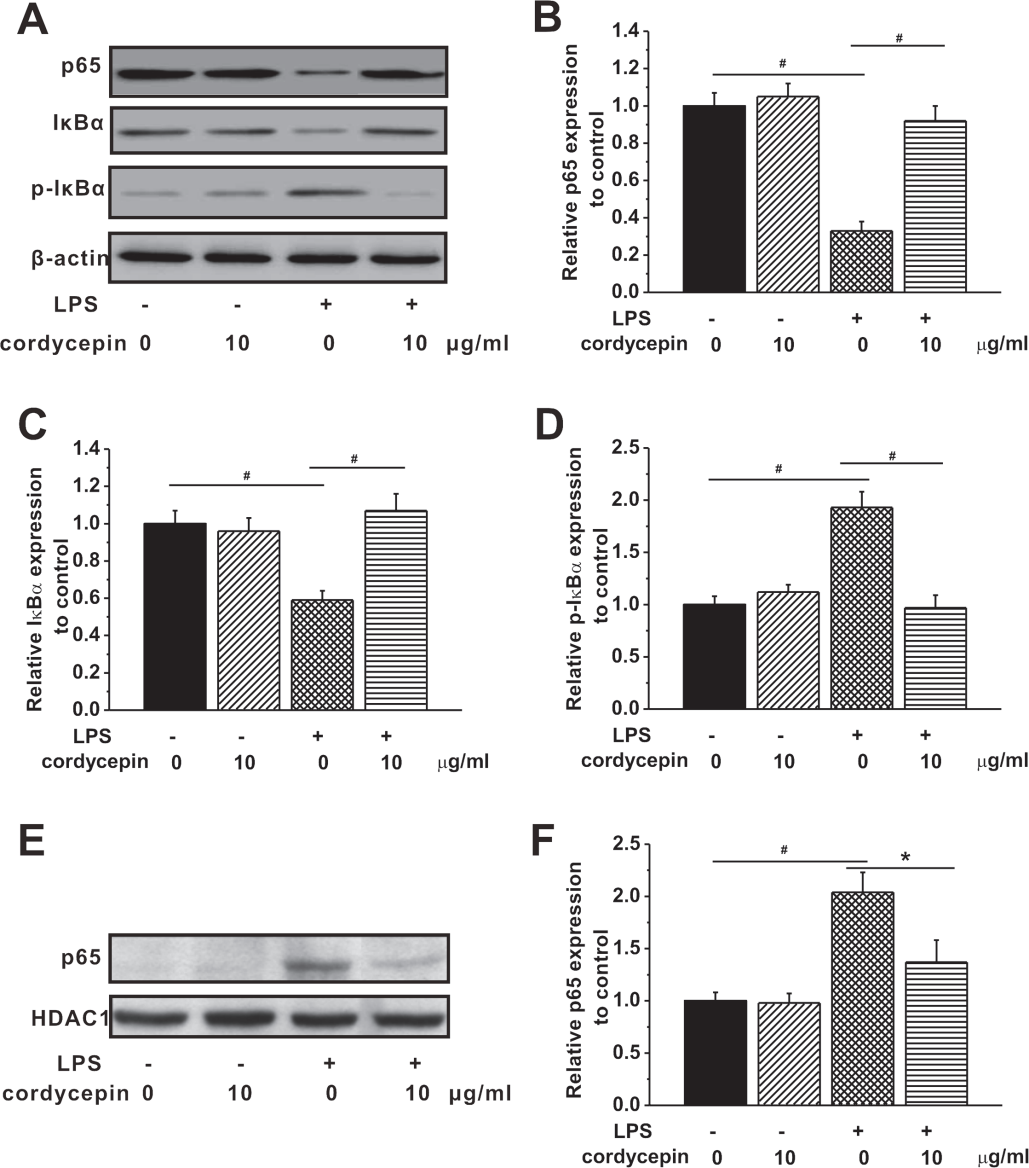
Cordycepin (10 μg/ml) inhibited NF-кB signaling pathway activated by LPS treatment. (A) The p65, IкBα and p-IкBα protein expression in the cytoplasm in the cultures under cordycepin treatment with or without LPS stimulation. (B-D) The relative optical densities of p65, IкBα and p-IкBα protein bands to control, normalized to β-actin. (E) The p65 expression in the nucleus in the experimental groups. (F) The relative optical densities of p65 protein bands to control, normalized to HDAC1. Data were presented by mean ± SEM. *p < 0.05, #p < 0.01.

To further investigate the mechanism of anti-inflammatory effect of cordycepin, we examined the NF-κB signaling pathway during the cordycepin treatment. The expressions of p65, IκBα, and phosphorylated IκBα (p-IκBα) were measured. Fig 3A shows the representative images of protein bands measured by western blotting analysis, including p65, IκBα and p-IκBα in the cytoplasm, in the different experimental groups. Fig 3B, 3C and 3D illustrate the relative expression of p65, IκBα and p-IκBα in the cytoplasm normalized by β-actin. P65 expression was dramatically decreased in LPS-stimulated microglia, while it was significantly increased nearly to the level in control (without LPS and cordycepin treatment) by cordycepin treatment in LPS-stimulated group (Fig 3B). Similar results could be observed in the IκBα expression in the cytoplasm. It was significantly decreased by LPS stimulation compared to control. Cordycepin treatment strikingly elevated IκBα expression in the cytoplasm to a comparable level to control in the LPS-stimulated culture (Fig 3C). In contrast, the p-IκBα expression was significantly higher in the cultures after LPS stimulation compare to control group, while it was dropped to the level of control by cordycepin treatment in LPS-stimulated cultures (Fig 3D). Furthermore, the expression of p65 in the nucleus was also examined (Fig 3E and 3F). Higher level of p65 in the nucleus was found in LPS-stimulated microglia, accounting for the decrease of p65 in the cytoplasm. These results revealed the occurrence of the translocation of p65 from cytoplasm to nucleus and the activation of NF-κB in LPS-stimulated microglia. However, p65 level in the nucleus was decreased in LPS-stimulated microglia after cordycepin treatment.

NF-κB is well-known to play ubiquitous roles in inflammation and immunological processes and also control cell division and apoptosis. It is employed by several distinctive biological cellular activities including inhibiting bone formation and cellular responses to injury of the nervous system [40, 41]. The TNF pathway involved in inflammatory response is one of the most widely-known characterized NF-κB-dependent pathways. In the nervous system, TNF can be activated by NF-κB and binds to TNF receptors expressed on neurons and microglia. P50, RelA/p65 and IκBα are the three most important proteins to activate NF-κB. In fact, the unphosphorylated IκBα protein mainly maintains the inactivated states of NF-κB proteins, including p50, p52, RelA/p65, and RelB [41]. Phosphorylation of IκBα by IκB kinase (IKK) is thought to be the central part of NF-κB signaling pathway, which leads to the ubiquitination and degradation of IκBα and in turn releases NF-κB proteins [42]. The released NF-κB proteins then translocate from cytoplasm to the nucleus to regulate cellular activities [42]. It was documented that LPS-stimulated rats contained increased transcriptional activation of IκB in microglia throughout the brain [43]. In addition, multiple studies have proposed the involvement of NF-κB in the activation of microglia [44, 45], and provided the evidence of the role of NF-κB in LPS-stimulated inflammation and the relation between NF-κB and microglia. Our results clearly demonstrated the ability of cordycepin to inhibit NF-κB signaling pathway in the induction of LPS-stimulated inflammation. This is in agreement with a previous study which revealed that pre-treatment of cordycepin suppressed the LPS-induced translocations of p65 to nucleus and blocked the LPS-stimulated degradation of IκB-α in BV2 cells [7]. Taken together, cordycepin has a potent clinical application value for not only preventing but also treating neuro-inflammation.

### Cordycepin treatment rescued the LPS-CM-induced neuronal death

**Fig 4:**
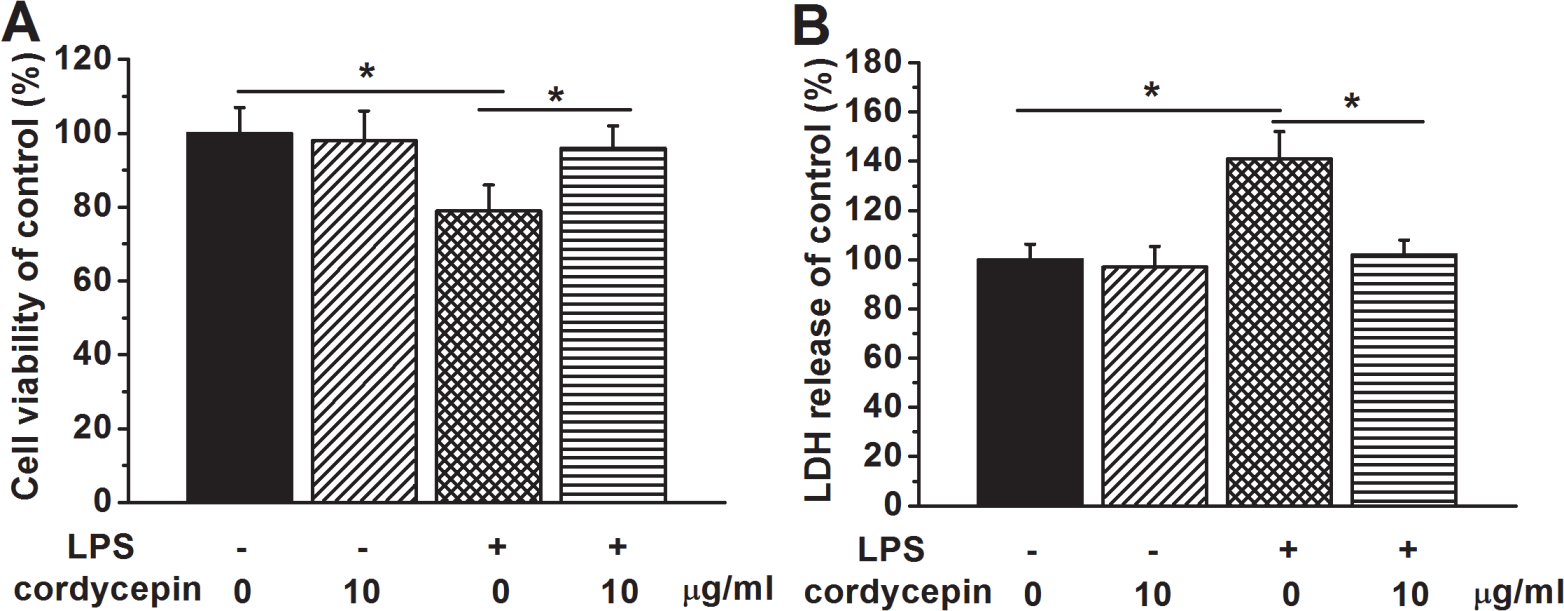
Cordycepin (10 μg/ml) rescued LPS-induced cell death in the hippocampal neurons. The cells were cultured for 7 days in the different conditioned mediums of the experimental groups. (A) Relative cell viabilities to control in the experimental groups, examined by MTT assay. (C) Relative LDH release to control in the experimental groups. Data were presented by mean ± SEM. *p < 0.05.

To investigate the beneficial effects of cordycepin on neural growth and development, we next examined the role of cordycepin in protecting neural death by LPS-induced microglial over-activation in the cultured hippocampal neurons. Firstly, we collected conditioned medium (CM) before performing turning experiments (see Methods and Materials section). CMs from microglia enriched cultures in the absence (control-CM) or presence of 10 μg/ml cordycepin (Cordycepin-CM) were prepared with (LPS-CM) or without LPS stimulation. Cell viability of the hippocampal neurons cultured in different CMs for 7 days was examined by MTT and LDH assay. The cell viability in the LPS-CM group was significantly declined compared to the control group (Fig 4A), indicating LPS-induced microglial over-activation may be harmful to the neurons. In contrast, 10 μg/ml cordycepin treatment greatly improved cell viability in the LPS-CM. LDH release results were also consistent with the above results (Fig 4B). Significantly higher level of LDH was released in LPS-CM cultured neurons in comparison with control group, whereas cordycepin treatment reversed this elevated LDH release, suggesting that the viability of neurons were improved. This was also verified in Aβ-stimulated situation (Fig. B in S1 File).

### Cordycepin protected the impairment of growth cone (GC) by microglial overactivation

**Fig 5:**
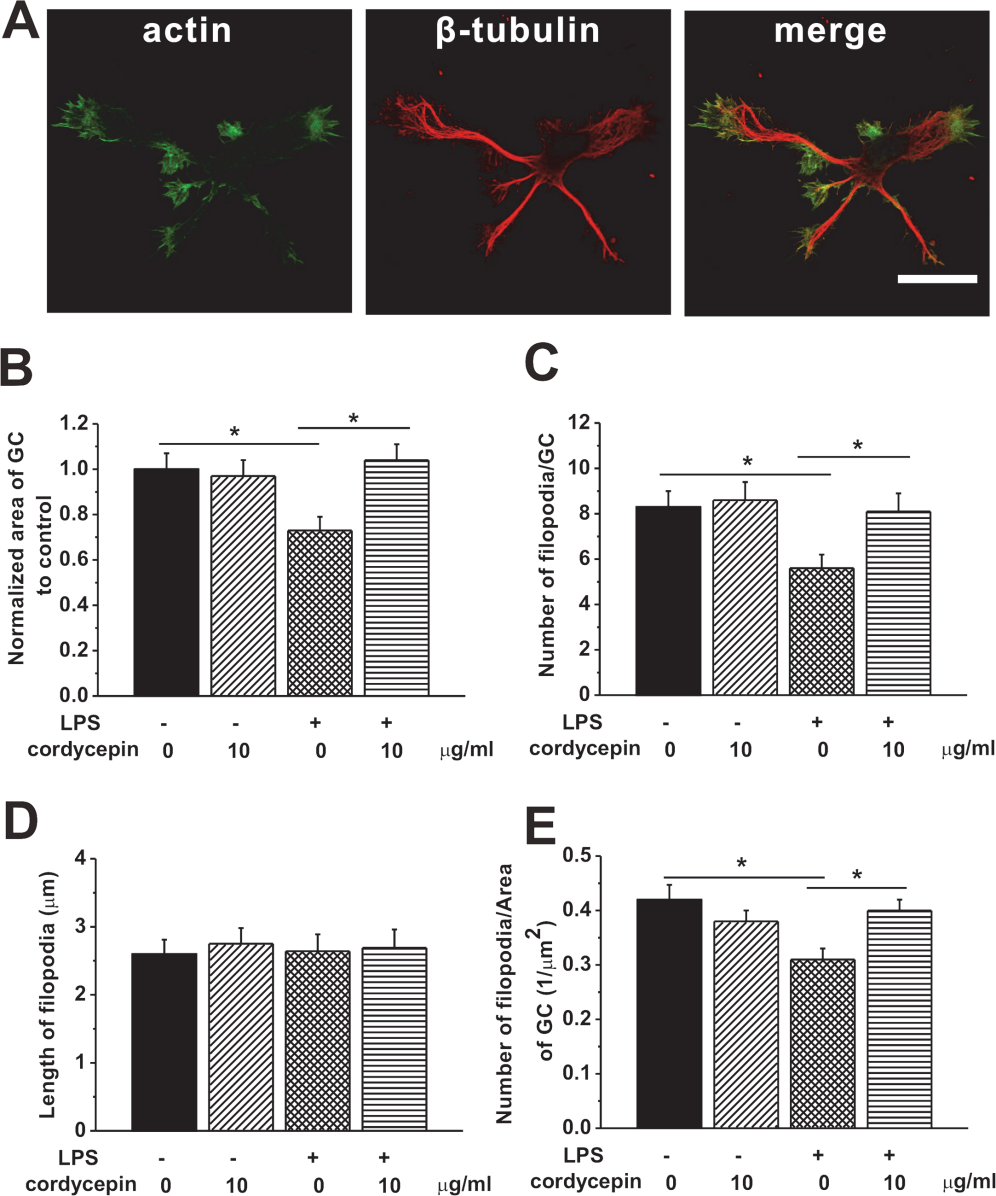
Cordycepin (10 μg/ml) rescued the LPS-induced impairments of GCs. The hippocampal neurons were cultured for 1 day in the different conditioned mediums of the experimental groups. (A) Representative fluorescence images of the hippocampal GC stained for actin, tubulin and merge of the two staining. Scale bar = 20 μm. (B) Relative area of GCs to control. (C) Average number of filopodia emerging from GCs. (D) Average filopodium lengths from the tip of each filopodia to the edge of the GCs. (E) Ratio of number of filopodia and area of GC in GCs. Data were presented by mean ± SEM. * p < 0.05.

Activated microglia increases the production of a variety of cytokines that have been shown to inhibit axonal growth [46–48]. Neurite exploration is guided by GCs located at their tip [49–51], formed by an extended lamellipodium from which thin filopodia emerge [52]. Firstly, we tried to determine the influence of cordycepin treatment on GCs extension and formation. The morphology of GCs was observed under fluorescence microscope by actin and β-tubulin labeling. Fig 5A shows representative images of GC with actin rich peripheral zone (green) and microtubule-rich central core (red). The normalized area of GC to control in the hippocampal cultures was significantly reduced by LPS-CM culturing, while the cordycepin treatment promisingly reversed this reduction (Fig 5B). Filopodia, which radiates out from the central core of GC, functions as the probe to detect the surrounding environment [53, 54]. The number of filopodia of each GC was significantly decreased in LPS-CM cultured neurons, and it can be rescued by cordycepin treatment as indicated by a significant increase in the number of filopodia, suggesting the impairments of inflammation on the development of neurons. However, cordycepin may repair this effect by promoting the formation of filopodia, accounting for the increase of the number of filopodia in each GC by cordycepin treatment. Although no change of filopodium length was observed between different CMs treatments groups, the density of filopodia (number of filopodia/GC area) was reduced in LPS-CM cultured neurons compared to control group. GC density was significantly improved by cordycepin treatment in LPS-CM cultured neurons. Since the primary function of filopodia is to sense the factors released by other cells in the surrounding environment and transduce the signals to their parent growth cone, the disruption of the growth of filopodia can be related to the deficits of axonal transport, resulting in various brain disorders such as chronic neurodegenerative disease [55]. These results verified the impairments of GC extension and filopodia density by microglial over-activation and the capability of cordycepin in protecting neural impairments by inflammation.

### Cordycepin promoted neurite sprouting and outgrowth impaired by the LPS-CM

**Fig 6:**
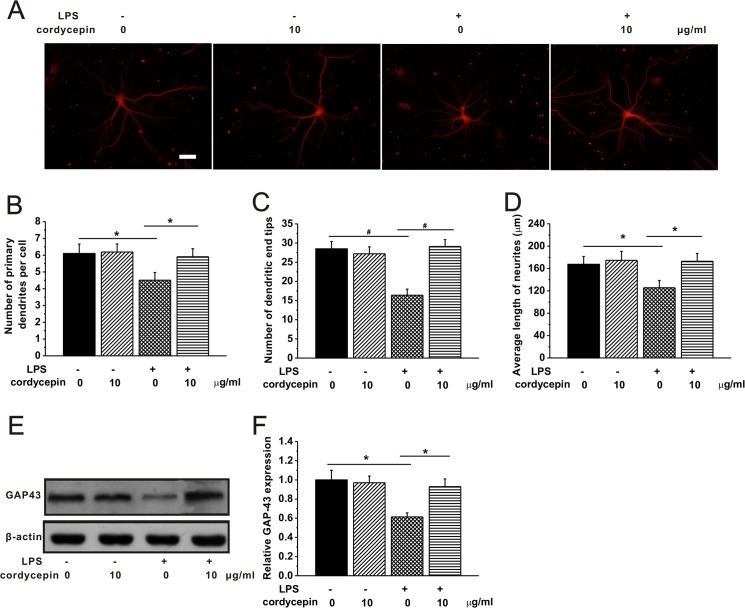
Cordycepin treatment (10 μg/ml) could repair the LPS-induced injuries of neurite sprouting and outgrowth. The hippocampal neurons were cultured for 7 day in the different conditioned mediums of the experimental groups. (A) Typical hippocampal neurons with extending neurites in the culture in different experimental groups, stained for MAP-2. The neurite sprouting and outgrowth were characterized by the number of primary dendrites per cell (B), number of dendritic end tips (C) and the average neurite length (D) in the experimental groups. (E) Western blot analysis of GAP-43 expression in the cultures of different groups. (F) Relative optical densities of GAP-43 show in (E) (n = 3/group). Data were presented by mean ± SEM. * p < 0.05, #p < 0.01.

**Fig 7:**
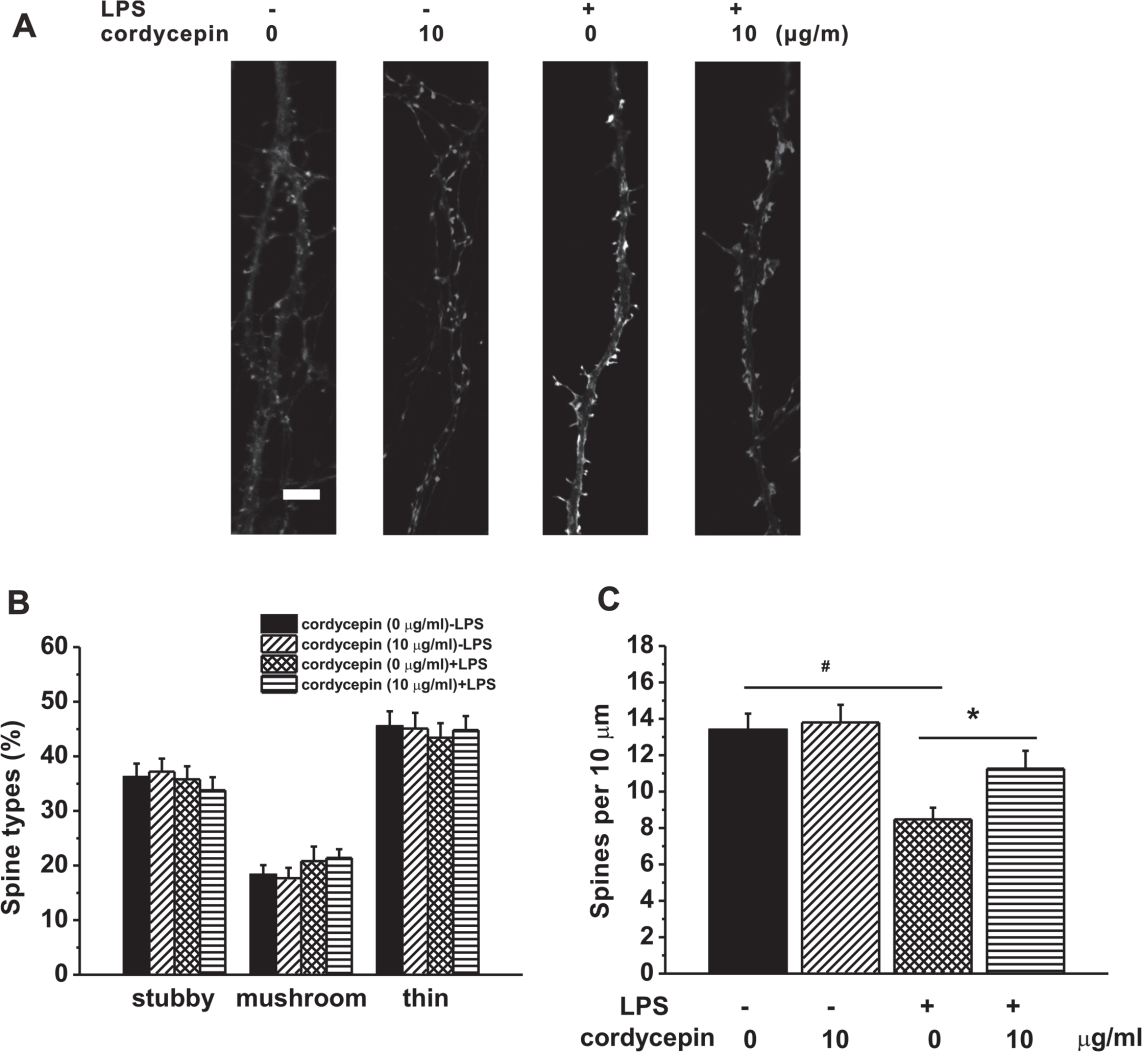
Effects of cordycepin treatment on spine morphology and density in the hippocampal neurons. (A) The cultured hippocampal neurons were transfected with mCherry-actin and imaged. The spine in the experimental groups could be clearly observed at high magnification. Scale bar = 2 μm. (B) Proportion of different spine types expressed as percentage of total spines in the neurons in the experimental groups. (C) Spine density expressed as number of spines per 10 μm of dendrites of the neurons in the experimental groups. Data were presented by mean ± SEM. * p < 0.05.

The neurons begun to extend neurites into the periphery after the neurons were seeded to the plates until they were mature. At day 7 after neurons were seeded, we studied the effects of cordycepin on the neurite sprouting and outgrowth by characterizing neurite density, length and complexity in the hippocampal neurons cultured in different CMs. Fig 6A shows typical hippocampal neurons with extending neurites stained by MAP-2, a maker for mature neurons, in different experimental groups. Significant decrease in the primary dendrites per neuron was observed in the LPS-CM cultured cells compared to control, while cordycepin significantly recovered the loss of neurites (Fig 6B). Similarly, LPS-CM could clearly reduce the numbers of dendritic end tips and the average length of neurites compared to control, indicating an inhibitory effects on neurite outgrowth (Fig 6C and 6D). As expected, cordycepin treatment exerted a protective role against the LPS-CM-induced impairments by promoting the number of dendritic tips as well as the averaged neurite length to a normal level of control (Fig 6C and 6D). These results provide strong evidences that cordycepin protects neurite sprouting and outgrowth in the event of microglial over-activation. We also measured the expression of growth associated protein 43 (GAP43), a protein considered to be a crucial factor involved in the neuronal development and plasticity [56, 57]. The expression of GAP43 in the cultures was significantly reduced in LPS-CM group compared to control (Fig 7E and 7F). As expected, cordycepin treatment led to a significant increase of GAP43 expression compared to LPS-CM group. This result further suggested that cordycepin is able to recover the impairments of neural growth and development after microglial over-activation.

### Effect of cordycepin on the spinogenesis

Finally, we examined the effect of cordycepin on spinogenesis by observing the changes of spine morphology and density in different cultures of CMs. The neurons were cultured for 17 days and were transfected with mCherry-actin. At day 21, the neurons with spines can be clearly visualized by confocal microscopy. Fig 7A shows the images of the neurite with spine in a higher magnification. The proportions of different spine types (stubby, mushroom, thin) were compared among the four experimental groups. No significant difference was observed among these groups (Fig 7B), indicating that neither LPS-CM nor cordycepin treatment can influence the spine morphology after the neurons matured. However, the spine density in the neurons cultured in LPS-CM was significantly lower compared to that of control (Fig 7C). Remarkably, increased spine density was also observed in the hippocampal neurons after cordycepin treatment.

Taken all together, these results provided supportive evidence of the protective role of cordycepin against the impairments of brain caused by neuroinflammation by inhibiting TNF-α, IL-1β, iNOS and COX-2. It was also indicated that neural development were facilitated by cordycepin treatment. Previous study demonstrated that 20 mg/kg cordycepin taken orally had the capacity to decrease plasma IL-1β and TNF-α in mice [58]. Moreover, a study on the role of cordycepin in cerebral ischema/reperfusion validated the potent neuroprotective function of cordycepin *in vivo* [6]. These studies suggest that cordycepin may penetrate brain and act as an anti-inflammatory agent in treating brain injury. However, if cordycepin is effective to be used in treating microglia-overactivation-induced inflammatory damage of brain still needs *in vivo* experiments in further study.

In conclusion, we report here that cordycepin possess anti-inflammatory effects in LPS-induced microglial activation *via* inhibiting NF-κB signaling pathway. Cordycepin treatment is able to recover the neuronal death caused by microglial over-activation and successfully rescue the inflammation-induced impairments of neural growth and development in the hippocampal cultured neurons, as indicated by improved growth cone extension, neurite sprouting and outgrowth and spinogenesis. Our study demonstrates the neuroprotective role of cordycepin against neuroinflammation-induced impairments and suggests the potential of cordycepin as an alternative therapeutic approach to treat brain injuries.

## Supporting Information

S1 File Combined File. Fig. A, 5 μM Amyloid-beta (Aβ) treatment for 24 h induced less TNF-α (A) and IL-1β (B) release in the microglia, and 10 μg/ml cordycepin significantly reduced the release of TNF-α and IL-1β in Aβ-treated microglia. Data were presented by mean±SEM. * p < 0.05. Fig. B, 10 μg/ml cordycepin could rescue Aβ-CM-induced cell death in the hippocampal neurons. The cells were cultured for 7 days in the different CMs. (A) Relative cell viability to control, examined by MTT assay. (B) Relative LDH release to control. Data were presented by mean±SEM. * p < 0.05.
(DOCX)
