# Supplementary material for: Effects of Cordycepin on the Microglia-Overactivation-Induced Impairments of Growth and Development of Hippocampal Cultured Neurons
Source: PLoS One. 2015 May 1;10(5):e0125902. doi: 10.1371/journal.pone.0125902 (PMC4416906; doi:10.1371/journal.pone.0125902)
Supplement: S1 File — Data were presented by mean±SEM. * p < 0.05. Fig. B, 10 μg/ml cordycepin could rescue Aβ-CM-induced cell death in the hippocampal neurons. The cells were cultured for 7 days in the different CMs. (A) Relative cell viability to control, examined by MTT assay. (B) Relative LDH release to control. Data were presented by mean±SEM. * p < 0.05. (DOCX) [file pone.0125902.s001.docx]

**Supporting Information**

**Supplementary Figures**





**Fig A.** 5 µM Amyloid-beta (Aβ) treatment for 24 h induced less TNF-α (A) and IL-1β (B) release in the microglia, and 10 µg/ml cordycepin significantly reduced the release of TNF-α and IL-1β in Aβ-treated microglia. Data were presented by mean±SEM. * p < 0.05.





**Fig B.** 10 µg/ml cordycepin could rescue Aβ-CM-induced cell death in the hippocampal neurons. The cells were cultured for 7 days in the different CMs. (A) Relative cell viability to control, examined by MTT assay. (B) Relative LDH release to control. Data were presented by mean±SEM. * p < 0.05.
